# Effect of Antibiotic Resistance of Pathogens on Initial Antibiotic Therapy for Patients With Cholangitis

**DOI:** 10.7759/cureus.18449

**Published:** 2021-10-03

**Authors:** Sakue Masuda, Kazuya Koizumi, Haruki Uojima, Karen Kimura, Takashi Nishino, Junichi Tasaki, Chikamasa Ichita, Akiko Sasaki

**Affiliations:** 1 Gastroenterology Medicine Center, Shonan Kamakura General Hospital, Kanagawa, JPN

**Keywords:** post-ercp cholecystitis, risk factors, endoscopic retrograde cholangiopancreatography, cholangitis, antibiotic-resistant pathogens, antibacterial agents

## Abstract

Objectives

Considering that pathogens resistant to initial antibiotic therapies for cholangitis can affect mortality rates, appropriate initial empiric antibiotic therapy is important. However, evidence regarding the influence of pathogens resistant to initial antibiotics in patients with cholangitis who have undergone early endoscopic retrograde cholangiopancreatography (ERCP) is limited, and the conditions in several cases can improve with early ERCP even when pathogens resistant to initial antibiotics are detected on time. Therefore, this study aimed to assess the influence of pathogens resistant to initial antibiotics on the course of cholangitis in patients undergoing early ERCP.

Materials and methods

Patients (n=266) with positive blood or bile culture results treated with early ERCP were divided into those with cultures that were resistant to the initial antibiotics (antibiotic-resistant group; n=66; 24.8%) and those with cultures that were sensitive to the initial antibiotics (antibiotic-sensitive group; n=200; 75.2%). The duration of hospitalization, in-hospital mortality rates due to cholangitis, rates of increased disease severity, and complications during hospitalization were studied.

Results

*Enterococcus*, *Enterobacter*, *Citrobacter*, and *Pseudomonas* species showed high resistance to several antibiotics. No significant between-group differences were found in the duration of hospitalization, in-hospital mortality rates due to cholangitis, and rates of increased disease severity. However, the rate of post-ERCP cholecystitis was significantly higher in the antibiotic-resistant group than in the antibiotic-sensitive group (p=0.0245).

Conclusions

Even if the initial antibiotics were ineffective, the rate of fatal outcomes did not increase among patients with cholangitis who had undergone early ERCP. However, when initial antibiotics were ineffective, the frequency of post-ERCP cholecystitis increased even after early bile duct decompression.

## Introduction

Cholangitis is the second most common cause of community-acquired bacteremia and bacteremia in older patients [1-2]. Cholangitis-related mortality rates are relatively high at 5%-10% [3-4], particularly among older patients [5-6]. Some studies have suggested that infection caused by pathogens resistant to the initial antibiotic therapy in patients with cholangitis is a predictor of mortality [7-8]. Therefore, appropriate initial empiric antibiotics should be administered to ensure favorable clinical outcomes. The recommended initial empiric antibiotics are typically administered to patients with cholangitis depending on the risk category, and treatment is tailored according to the susceptibility results of the culture. The Tokyo Guidelines 2018 for cholangitis recommend initial broad-spectrum antibiotics, even for mild or moderate cholangitis. However, evidence for appropriate initial empiric antibiotics is insufficient [9].

Conversely, early endoscopic retrograde cholangiopancreatography (ERCP) performed within 48 or 72 h may improve patient outcomes [10-12]. A previous study showed that infection with pathogens resistant to the initial antibiotics was not significantly associated with increased mortality in patients with cholangitis undergoing therapeutic drainage [7]. These findings reflect the importance of early biliary decompression and general supportive care [11,13]. Therefore, initial antibiotics, even if ineffective, may not affect the prognosis in cases where early therapeutic drainage is successful. Thus, this study aimed to assess the effect of pathogens resistant to the initial antibiotics on the course of patients with cholangitis undergoing early ERCP.

## Materials and methods

Study population and sample size

**Figure 1:**
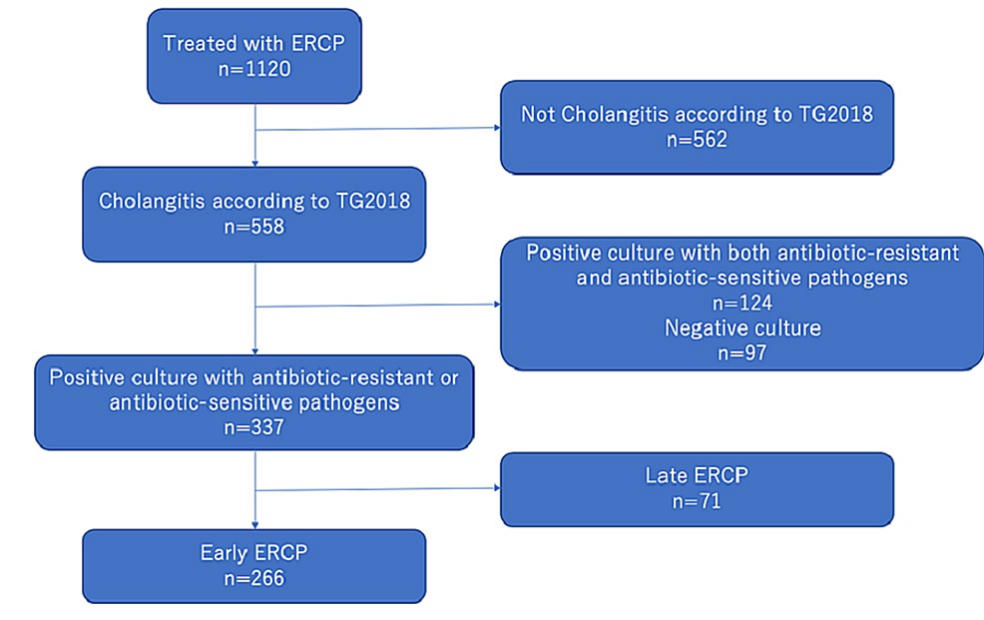
Study population The hospital records of patients treated from April 2018 to March 2020 were searched to identify patients with cholangitis who had positive blood or bile cultures and underwent ERCP within 24 h after being first checked by the physician. ERCP, endoscopic retrograde cholangiopancreatography; TG2018, Tokyo Guidelines 2018

This retrospective observational cohort study was conducted at the Shonan Kamakura General Hospital in Japan. Hospital records of patients treated from April 2018 to March 2020 were searched to identify patients with cholangitis who had positive blood or bile cultures and underwent ERCP within 24 h after the first physician contact. In principle, blood cultures were collected before antibiotic administration, and bile cultures were collected immediately after the start of ERCP. ERCP was performed urgently in severe cases and on the following day in mild cases. Patients who underwent late ERCP were excluded, and those with cultures positive for both antibiotic-resistant and antibiotic-sensitive pathogens were also excluded (Figure 1). This was performed because it is difficult to determine whether an antibiotic agent is effective or ineffective in the course of cholangitis if it is effective only against some of the causative organisms.

When the primary outcome was the duration of hospitalization, under the conditions of a difference between the two groups of one day, a standard deviation value of two days, a patient ratio of 1:3, two-sided α=0.05, and 1-β=0.90, 57 patients with antibiotic-resistant pathogen infections and 172 with antibiotic-sensitive pathogen infections were required, as per sample size calculation [14]. Since we used a nonparametric test to compare the length of hospitalization, we increased the sample size by about 10%.

Ethical information

All procedures were performed in accordance with the ethical standards established in the 1964 Declaration of Helsinki and its later amendments. The study was reviewed and approved by the institutional review board of the Future Medical Research Center Ethical Committee (IRB no. TGE00956-024). Owing to the retrospective study design, informed consent was obtained from all participants by the opt-out method on our hospital website and in-hospital posting.

Study variables

This study aimed to assess the effect of infection caused by pathogens resistant to the initial antibiotics on the course of patients with cholangitis undergoing early ERCP. The following data were collected from the medical records: age, sex, underlying disease, cause of infection, severity of infection, laboratory results, causative microorganisms, antibiotic susceptibility, administration of inappropriate antibiotics, and ERCP findings. Outcomes included the duration of hospitalization, mortality during hospitalization, increased disease severity, and complications during hospitalization.

In our hospital, ampicillin/sulbactam, cefmetazole, ceftriaxone, piperacillin/tazobactam, meropenem, and ciprofloxacin are typically used as initial treatment. In mild cases, cefmetazole was administered primarily, and other antibiotics were administered with reference to renal function and previous cultures. In severe cases, primarily piperacillin/tazobactam or meropenem was administered.

If plastic stent implantation is needed in ERCP, a single 7-Fr stent was implanted as a rule. In self-expandable metallic stent (SEMS), stents with 10- and 8-mm diameters were implanted in the common bile duct and hilar region, respectively.

Definitions

The diagnosis and severity of cholangitis were based on the Tokyo Guidelines 2018 [15]. Pathogen resistance to the initial antibiotics was defined as a pathogen that is resistant to the initial antibiotics in vitro. Cholangitis is often caused by polymicrobial infection. Blood culture has low sensitivity and may not detect the causative organism, whereas bile culture has low specificity and may detect enteric bacteria that are not the cause of the inflammation, resulting in difficulty in the accurate identification of causative organisms. Therefore, in this study, all detected bacteria were equally considered causative organisms. The term “appropriate definitive antibiotics” was defined as antibiotics with in vitro activity against the isolated microorganisms. Early ERCP was defined as ERCP performed within 24 h after the first physician contact during hospitalization. The clinical success of ERCP was defined as a decrease in the total bilirubin or alanine aminotransferase level by one-half or normalization within one week of ERCP.

Statistical analyses

The Mann-Whitney U-test was used to compare non-normally distributed continuous variables, and the χ2 test or Fisher’s exact test was used to compare categorical variables. Multivariate analysis was performed using logistic regression. Variables that were clinically significant or reported in previous studies were included in the multivariate analysis. Two-tailed p-values <0.05 were considered significant. All statistical analyses were performed using EZR (Saitama Medical Center, Jichi Medical University, Saitama, Japan), which is a graphical user interface for R (The R Foundation for Statistical Computing, Vienna, Austria). More precisely, it is a modified version of R Commander designed to allow additional statistical functions frequently used in biostatistics [16].

## Results

Patient characteristics

Table 1 summarizes the characteristics of the patients. Data from 266 patients with positive blood or bile cultures who were treated with early ERCP were retrospectively analyzed. Of these patients, 66 (24.8%) were infected with pathogens resistant to the initial antibiotics (antibiotic-resistant group) and 200 (75.2%) were infected with a pathogen sensitive to the initial antibiotics (antibiotic-sensitive group), with no significant between-group differences with regard to age, sex, cause of cholangitis, severity, underlying disease, or patient background.

**Table 1:** Patient characteristics † Some patients had more than one underlying disease. CKD, chronic kidney disease; CHF, chronic heart failure; LC, liver cirrhosis; DM, diabetes mellitus

	Antibiotic-resistant	Antibiotic-sensitive	p
	n=66 (24.8%)	n=200 (75.2%)	
Age, years	80 (33–97)	78 (25–102)	0.568
Sex	Male, 34; female, 32	Male, 113; female, 87	0.57
Cause of cholangitis			
Malignant stricture	19 (28.8%)	61 (30.5%)	0.877
Bile duct stone	45 (68.2%)	131 (65.5%)	0.765
Benign bile duct stricture	1 (1.5%)	4 (2.0%)	>0.99
Chronic pancreatitis	0 (0%)	2 (1.0%)	>0.99
Others	1 (1.5%)	2 (1.0%)	>0.99
Severity			
Mild	27 (40.9%)	84 (42.0%)	>0.99
Moderate	34 (51.5%)	82 (41.0%)	0.153
Severe	5 (7.6%)	34 (17.0%)	0.071
Underlying disease			
CKD	3 (4.5%)	6 (3.0%)	0.694
CHF	8 (12.1%)	18 (9.0%)	0.476
LC	3 (4.5%)	6 (3.0%)	0.694
DM	11 (16.7%)	35 (17.5%)	>0.99
Malignant tumor	20 (30.3%)	52 (26.0%)	0.524
Total^†^	34 (51.5%)	97 (48.5%)	0.777
Patient background			
Nursing home	13 (19.7%)	40 (20.0%)	>0.99
Hemodialysis	2 (3.0%)	0 (0%)	0.061
Gastrostomy	0 (0%)	0 (0%)	
Constant placement of a urinary catheter	0 (0%)	1 (0.5%)	>0.99
Aspiration pneumonia	0 (0%)	3 (1.5%)	>0.99
Immunosuppressant	0 (0%)	9 (4.5%)	0.118
Bile duct stent re-intervention	11 (16.7%)	38 (19.0%)	0.719

Microbiological data

The laboratory findings of the microbial cultures from the patients are summarized in Table 2. The positivity rates for detecting microorganisms in the blood and bile cultures were 54.5% (105/193) and 99.2% (259/261), respectively. A total of 147 (55.3%) patients had polymicrobial infections, for which 434 microorganisms were detected. Escherichia coli, Klebsiella species, Enterococcus species, and Enterobacter species were the most common pathogens. Enterococcus species, Enterobacter species, Citrobacter species, Pseudomonas species, and Aeromonas showed high resistance to many antibiotics. Especially, the resistance rates of Enterococcus species to most antibiotics were ≥20%. In general, when ≥20% of microorganisms display antibiotic resistance, the antibiotic used is ineffective as a first-line empiric agent.

**Table 2:** Antibiotic resistance of isolated bacteria based on culture sensitivity testing

Bacteria isolated in culture	Number of species (% of total)	Resistant against						
		Ampicillin/ sulbactam	Piperacillin/ tazobactam	Ceftriaxone	Cefmetazole	Carbapenem	Ciprofloxacin	Levofloxacin
Escherichia coli	112 (25.8%)	18 (16.1%)	1 (0.9%)	11 (9.8%)	2 (1.8%)	0 (0%)	13 (11.6%)	13 (11.6%)
Klebsiella species	90 (20.7%)	9 (10%)	0 (0%)	0 (0%)	0 (0%)	0 (0%)	0 (0%)	0 (0%)
Enterococcus species	71 (16.4%)	12 (16.9%)	15 (21.1%)	71 (100%)	71 (100%)	20 (28.2%)	20 (28.2%)	17 (23.9%)
faecalis	28 (6.5%)	0 (0%)	1 (3.6%)	28 (100%)	28 (100%)	3 (10.7%)	3 (10.7%)	3 (10.7%)
faecium	16 (3.7%)	10 (62.5%)	11 (68.8%)	16 (100%)	16 (100%)	5 (93.8%)	12 (75.0%)	12 (75.0%)
avium	3 (0.7%)	2 (66.7%)	3 (100%)	3 (100%)	3 (100%)	2 (66.7%)	1 (33.3%)	1 (33.3%)
casseliflavus	18 (4.1%)	0 (0%)	0 (0%)	18 (100%)	18 (100%)	0 (0%)	3 (16.7%)	1 (5.6%)
others	5 (1.2%)	0 (0%)	0 (0%)	5 (100%)	5 (100%)	0 (0%)	1 (20.0%)	0 (0%)
Enterobacter species	43 (9.9%)	32 (74.4%)	3 (7.0%)	6 (14.0%)	37 (86%)	0 (0%)	1 (2.3%)	0 (0%)
Citrobacter species	10 (2.3%)	10 (83.3%)	3 (25.0%)	5 (41.7%)	10 (83.3%)	0 (0%)	1 (8.3%)	0 (0%)
Staphylococcus	7 (1.6%)	1 (14.3%)	1 (14.3%)	2 (28.6%)	1 (14.3%)	1 (14.3%)	3 (42.9%)	2 (28.6%)
Streptococcus	36 (8.3%)	3 (8.3%)	5 (13.9%)	0 (0%)	10 (27.7%)	0 (0%)	8 (22.2%)	4 (11.1%)
Pseudomonas species	11 (2.5%)	11 (100%)	1 (9%)	11 (100%)	11 (100%)	1 (9%)	0 (0%)	0 (0%)
Aeromonas	18 (4.1%)	18 (100%)	0 (0%)	5 (27.8%)	6 (33.3%)	0 (0%)	1 (5.5%)	0 (0%)
Clostridium	8 (1.8%)	0 (0%)	0 (0%)	0 (0%)	0 (0%)	0 (0%)	0 (0%)	0 (0%)
Bacteroides	1 (0.2%)	0 (0%)	0 (0%)	1 (100%)	0 (0%)	0 (0%)	0 (0%)	0 (0%)
Others	23 (5.3%)	2 (8.7%)	0 (0%)	2 (8.7%)	1 (4.3%)	0 (0%)	2 (8.7%)	2 (8.7%)
Total	434	116 (26.7%)	29 (6.7%)	114 (26.3%)	149 (34.3%)	22 (5.0%)	49 (11.3%)	38 (8.8%)

ERCP findings

Table 3 summarizes the ERCP findings of the study population. The median time to ERCP was four (2-24) h in the antibiotic-resistant group and four (1-24) h in the antibiotic-sensitive group. The technical success rates of ERCP were 100% and 98% (196/200) in the antibiotic-resistant and antibiotic-sensitive groups, respectively. Unsuccessful cannulation was because of Roux-en-Y reconstruction (n=3) and ampullary cancer (n=1). Of the four patients in whom ERCP was unsuccessful, three underwent endoscopic ultrasonography-guided hepaticogastrostomy, and one patient did not undergo any biliary drainage procedure. The clinical success rate of biliary drainage was 92.2% in both groups. No significant between-group differences were found with regard to the time to ERCP, rate of prior ERCP, the clinical success rate of biliary drainage, or complications other than cholecystitis.

**Table 3:** ERCP findings and antibiotic therapy † One patient had two complications. ‡ Judgment could not be made in two cases because of insufficient data. ⁋ Judgment could not be made in seven cases because of insufficient data. *Excluding cases that were difficult to evaluate as post-ERCP cholecystitis such as post-cholecystectomy, gallbladder cancer, and cholecystitis on admission. ERCP, endoscopic retrograde cholangiopancreatography; ENBD, endoscopic nasobiliary drainage; SEMS, self-expandable metallic stent

			Antibiotic-resistant			Antibiotic- sensitive			p			
			n=66 (24.8%)			n=200 (75.2%)						
ERCP findings												
Median time from first physician contact to ERCP (h) (range)				4 (2–24)		4 (1–24)			0.0983			
Prior ERCP				23 (34.8%)		79 (39.2%)			0.561			
Technical success of ERCP				100%		98%			0.575			
Clinical success of biliary drainage				92.2%^‡^		92.2%^⁋^			>0.99			
ERCP drainage procedure												
Stent replacement				26 (39.4%)		102 (51.0%)			0.119			
SEMS				9 (13.6%)		21 (10.5%)			0.504			
Plastic stent				17 (25.8%)		81 (40.5%)			0.039			
ENBD				5 (7.6%)		14 (7.0%)			>0.99			
Lithotripsy				38 (57.6%)		83 (41.5%)			0.032			
Complications												
Pancreatitis				0 (0%)		6 (3.0%)			0.341			
Bleeding				3 (4.5%)		4 (2.0%)			0.37			
Perforation				0 (0%)		1 (0.5%)			>0.99			
Cholecystitis*				5 (10.0%)		3 (1.9%)			0.025			
Stent migration/early stent obstruction				1 (1.4%)		0 (0%)			0.248			
Total				8 (12.1%)^†^		14 (7.0%)			0.202			
Antibiotic therapy												
Initial antibiotic therapy												
Ampicillin/sulbactam	38 (14.3%)				11 (16.7%)		27 (13.5%)			0.422		
Piperacillin/tazobactam	50 (18.8%)				1 (1.5%)		49 (24.5%)			<0.001		
Ceftriaxone	19 (7.1%)				11 (16.7%)		8 (4.0%)			0.001		
Cefmetazole	138 (51.9%)				38 (57.6%)		100 (50.0%)			0.321		
Carbapenem	8 (3.0%)				0 (0%)		8 (4.0%)			0.207		
Ciprofloxacin	7 (2.6%)				2 (3.0%)		5 (2.5%)			>0.99		
Levofloxacin	0 (0%)				0 (0%)		0 (0%)			1		
Others	4 (1.5%)				1 (1.5%)		3 (1.5%)			>0.99		
No antibiotic	2 (0.8%)				2 (3.0%)		0 (0%)			0.061		
Median time from first physician contact to antibiotic administration (range)					3.5 h (1–28)		3 h (0–29)			0.036		
Antibiotic administration within 6 h					48 (72.3%)		172 (86.0%)			0.015		
Median duration of antibiotic therapy (range)				5 days (0–24)			5 days (1–49)			0.101		
Change from initial antibiotic agent to appropriate definitive antibiotic therapy				15 (22.7%)								

Post-ERCP cholecystitis occurred in 10.0% (5/50) of patients in the antibiotic-resistant group and 1.9% (3/157) of patients in the antibiotic-sensitive group (p=0.0245), excluding cases that were difficult to evaluate as post-ERCP cholecystitis such as post-cholecystectomy, gallbladder cancer, and cholecystitis on admission. Cholecystitis was observed at a median of five days (3-8 days) after ERCP. The median age of the patients with cholecystitis was 88.5 (79-96) years. Of these patients, 50% (n=4) were treated with self-expandable metallic stents and 62.5% (n=5) were infected with pathogens resistant to the initial antibiotics. All cases showed improvement with drainage procedures such as endoscopic retrograde gall bladder drainage or endoscopic ultrasonography-guided gall bladder drainage.

Antibiotic therapy and microorganisms in each group

Tables 3-4 summarize the microbiological findings of the study. The antibiotics administered for the initial treatment included cefmetazole (n=138; 51.9%), piperacillin/tazobactam (n=50; 18.8%), ampicillin/sulbactam (n=38; 14.3%), ceftriaxone (n=19; 7.1%), carbapenems (n=8; 3.0%), ciprofloxacin (n=7; 2.6%), and others (n=4, 1.5%). Two of the patients (0.8%) did not receive antibiotics.

**Table 4:** Microorganisms and clinical outcomes † A total of 147 (55.3%) patients had more than one pathogen. ESBL, extended-spectrum β-lactamase

			Antibiotic-resistant		Antibiotic-sensitive		p
			n=66 (24.8%)		n=200 (75.2%)		
*Microorganisms in each group* ^†^							
Microorganisms							
Escherichia coli			8 (12.1%)			104 (52.0%)	<0.001
ESBL			3 (4.5%)			7 (3.5%)	0.713
Klebsiella species			4 (6.1%)			86 (43.0%)	<0.001
Enterococcus species			32 (48.5%)			37 (18.5%)	<0.001
faecalis			12 (18.2%)			16 (8.0%)	0.035
faecium			10 (15.2%)			6 (3.0%)	0.001
avium			2 (3.0%)			1 (0.5%)	0.153
casseliflavus			6 (9.1%)			12 (6.0%)	0.401
Others			3 (3.0%)			3 (1.5%)	0.6
Enterobacter species			21 (31.8%)			22 (11.0%)	<0.001
Citrobacter species			7 (10.6%)			5 (2.5%)	0.012
Staphylococcus			1 (1.5%)			6 (3.0%)	>0.99
Streptococcus			8 (12.1%)			28 (14.0%)	0.837
Pseudomonas species			8 (12.1%)			3 (1.5%)	<0.001
Aeromonas			3 (4.5%)			15 (7.5%)	0.575
Clostridium			0 (0%)			8 (4.0%)	0.207
Bacteroides			0 (0%)			1 (0.5%)	>0.99
Others			2 (3.9%)			21 (10.5%)	0.076
Clinical outcomes							
Duration of hospitalization (days)							
All cases		7 days (3–67)		7.5 days (2–50)			0.707
Severe cases		10 days (10–15)		9 days (2–33)			0.229
Mild or moderate cases		7 days (3–67)		7 days (3–50)			0.752
In-hospital mortality due to cholangitis		2 (3.0%)		3 (1.5%)			0.6
Increased severity in mild and moderate cases		10 (16.4%)		41 (24.7%)			0.212

The median times from the first physician contact to antibiotic administration were 3.5 h and 3 h in the antibiotic-resistant group and antibiotic-sensitive group, respectively. The median duration of antibiotic therapy was five days in both groups.

In the antibiotic-resistant group, 15 (22.7%) patients received appropriate definitive antibiotics, in which the initial antibiotic agent was changed, and 41 (77.3%) patients discontinued the initial antibiotics. The initial antibiotic agent was changed because of increased disease severity, persistent low-grade fever, persistently increased C-reactive protein level or leukocyte count, and ineffective biliary drainage. Antibiotics were discontinued when the patient no longer had a fever, leukocyte count began to decline, and biliary drainage was effective (Table 3).

In the antibiotic-resistant group, Enterococcus species and Enterobacter species were the most common pathogens, and Enterococcus species, Enterobacter species, Citrobacter species, and Pseudomonas species were detected significantly more frequently in the antibiotic-resistant group than in the antibiotic-sensitive group (Table 4).

Clinical outcomes

No significant between-group differences in the duration of hospitalization, in-hospital mortality rates due to cholangitis, and rates of increased disease severity were observed (Table 4). However, the rate of post-ERCP cholecystitis was significantly higher in the antibiotic-resistant group than in the antibiotic-sensitive group (p=0.0245).

Multivariate analysis for post-ERCP cholecystitis

Multivariate analysis showed that pathogens resistant to the initial antibiotics (odds ratio [OR] 8.39, 95% confidence interval [CI] 1.44-49.0, p=0.0182) and SEMS (OR 24.9, 95% CI 3.64-171.0, p=0.00105) independently predicted post-ERCP cholecystitis (area under the receiver operating characteristic curve [AUC ROC], 0.895 [>0.7]; multicollinearity <5) (Table 5).

**Table 5:** Multivariate analysis for post-ERCP cholecystitis ERCP, endoscopic retrograde cholangiopancreatography; CT, computed tomography; CI, confidence interval; HU, Hounsfield units

	Post-ERCP cholecystitis n=8	Non-post-ERCP cholecystitis n=199	Univariate analysis, p-value	Multivariate analysis, p-value	Odds ratio	95% CI
Age (years), median (range)	88 (76-91)	80.5 (33-102)	0.075			
Gallbladder stone	4 (50.0%)	105 (53.3%)	>0.99			
Gallbladder concentration (CT >10 HU)	6 (75.0%)	78 (39.4%)	0.065			
Gallbladder concentration (CT >20 HU)	3 (37.5%)	22 (11.1%)	0.059			
History of acute pancreatitis	0 (0%)	11 (5.6%)	>0.99			
Resistance against antibiotics	5 (62.5%)	45 (22.7%)	0.022	0.021	6.48	1.33–31.6
Biliary duct metallic stent	5 (62.5%)	21 (10.6%)	<0.001	<0.001	15.6	3.23–75.7
Tumor involvement to the orifice of the cystic duct	1 (12.5%)	13 (6.6%)	0.436			
Common bile duct diameter	4 (50.0%)	95 (48.0%)	>0.99			

## Discussion

Previous studies have revealed that infection with pathogens resistant to the initial antibiotics is independently associated with higher mortality of patients with cholangitis [17-18]. By contrast, another study showed that infection with pathogens resistant to the initial antibiotics was not significantly associated with increased mortality in patients with therapeutic drainage [7]. Delayed biliary decompression is regarded as the best-known predictor of poor outcomes [19], and the aforementioned findings probably reflect the importance of general supportive care and biliary decompression [13]. In the present study, infections with pathogens resistant to the initial antibiotics did not increase the incidence of fatal outcomes in patients with cholangitis who underwent early ERCP.

While no firm recommendation clarifying the timing of ERCP for patients with cholangitis exists [20], a recent study indicated that ERCP performed at >72 h after admission was associated with significantly higher rates of persistent organ failure and death than that performed at <72 h [11]. In another study, patients who underwent ERCP >48 h after admission exhibited a higher organ failure rate than those who underwent ERCP <48 h [10]. In many cases, we could provide ERCP within 24 h; under such conditions, fatal outcomes were not more frequent in the antibiotic-resistant group than in the antibiotic-sensitive group. Therefore, where possible, we recommend ERCP within 24 h for patients with cholangitis.

The duration of hospitalization, mortality rates due to cholangitis, and aggravation of cholangitis were not significantly different between the two groups; nevertheless, post-ERCP cholecystitis was more frequent in the antibiotic-resistant group than in the antibiotic-sensitive group (10.0% vs. 1.9%). A previous report observed post-ERCP cholecystitis in 1.35% of patients, and the risk factors included a history of acute pancreatitis and chronic cholecystitis, gallbladder opacification, and high leukocyte count before ERCP [21]. SEMS and tumor involvement at the orifice of the cystic duct have also been reported as risk factors for post-ERCP cholecystitis [22]. However, pathogens resistant to the initial antibiotics were not investigated as risk factors in previous reports. Our multivariate analysis showed that pathogens resistant to the initial antibiotics might also be a risk factor for post-ERCP cholecystitis. This is a novel finding of our study. In a previous study, SEMS was reported to be involved in post-ERCP cholecystitis in 55.6% of cases, and nearly half of the cases did not describe obstruction of the cystic duct [21]. In the present study, SEMS was involved in post-ERCP cholecystitis in 62.5% of the cases, which was comparable to that in the previous study; moreover, patients with post-ERCP cholecystitis tended to be older and to have CT values of 10 Hounsfield units (HU), 20 HU, or higher for gallbladder bile. Therefore, in the absence of SEMS, tumor involvement in the orifice of the cystic duct, and other obstructions of the cystic duct, the cause of post-ERCP cholecystitis may be an increase in pathogens resistant to the initial antibiotics due to bile congestion caused by poor gallbladder function.

Enterococcus, Enterobacter, Citrobacter, and Pseudomonas species were significantly more frequent in the antibiotic-resistant group. In general, these microorganisms were more common in patients with bile duct stent re-intervention and those dwelling in nursing homes. This was probably because an increased contact with healthcare workers and medical devices increases the risk of infection with pathogens resistant to antibiotics [4,23-29]. This is especially true for the Enterococcus species, which thrives in hospitals and is easily transmitted to nursing homes because of its inherent resistance to antibiotics and environmental stressors [24-25]. The initial colonizing strain of Enterococcus species can persist for several months on the surfaces of medical devices and in the gut [28-29]. This allows Enterococcus species to easily spread in hospitals and nursing homes. In the present study, we found that these antibiotic-resistant pathogens are a risk for post-ERCP cholecystitis. Because post-ERCP cholecystitis develops after a median of 4.5 days after ERCP and culture results are known by then, we can prepare for the development of cholecystitis if the culture results indicate the presence of resistant pathogens. Furthermore, we need to be aware of post-ERCP cholecystitis in patients who are at risk of exposure to resistant pathogens such as those who undergo bile duct stent re-intervention or those who reside in nursing homes. However, because we have not directly examined whether residing in a nursing home and re-intervention are risk factors for post-ERCP cholecystitis, further studies are needed to clarify these aspects.

This study has a few limitations. First, this was a retrospective study in a single tertiary referral center; therefore, some uncontrolled confounding factors might have affected the results. For example, we excluded patients who underwent ERCP more than 24 hours after the first physician contact and those who were culture positive for both antibiotic-resistant and antibiotic-sensitive pathogens, which may have lead to selective bias. Second, we cannot completely exclude the possibility that there were differences in the patient backgrounds because we did not evaluate patients using the American Society of Anesthesiologists classification of organ damage scoring. Finally, there could be variations in the skill of the endoscopist performing ERCP.

## Conclusions

Even if the initial antibiotic was not effective, the rates of fatal outcomes were not increased among patients with cholangitis who underwent early ERCP. However, when the initial antibiotic was not effective, the frequency of post-ERCP cholecystitis increased even after early bile duct decompression. Hence, initial broad-spectrum antibiotic therapy (e.g. piperacillin/tazobactam or carbapenems [and vancomycin for patients with risk factors for E. faecium infection]) should be considered for patients with bile duct stent re-intervention and those dwelling in nursing homes, which are risk factors for pathogens resistant to initial antibiotics. However, because we have not directly examined whether residing in a nursing home and re-intervention are risk factors for post-ERCP cholecystitis, further studies are needed to clarify these aspects.
